# The Formation of Biofilms by *Pseudomonas aeruginosa*: A Review of the Natural and Synthetic Compounds Interfering with Control Mechanisms

**DOI:** 10.1155/2015/759348

**Published:** 2015-03-19

**Authors:** Tsiry Rasamiravaka, Quentin Labtani, Pierre Duez, Mondher El Jaziri

**Affiliations:** ^1^Laboratory of Plant Biotechnology, Université Libre de Bruxelles, rue des Professeurs Jeener et Brachet 12, 6041 Gosselies, Belgium; ^2^Department of Therapeutical Chemistry and Pharmacognosy, Université de Mons, Avenue Maistriau 19, Bâtiment Mendeleiev, 7000 Mons, Belgium

## Abstract

*P. aeruginosa* is an opportunistic pathogenic bacterium responsible for both acute and chronic infections. Beyond its natural resistance to many drugs, its ability to form biofilm, a complex biological system, renders ineffective the clearance by immune defense systems and antibiotherapy. The objective of this report is to provide an overview (i) on *P. aeruginosa* biofilm lifestyle cycle, (ii) on the main key actors relevant in the regulation of biofilm formation by *P. aeruginosa* including QS systems, *GacS*/*GacA* and *RetS*/*LadS* two-component systems and C-di-GMP-dependent polysaccharides biosynthesis, and (iii) finally on reported natural and synthetic products that interfere with control mechanisms of biofilm formation by *P. aeruginosa* without affecting directly bacterial viability. Concluding remarks focus on perspectives to consider biofilm lifestyle as a target for eradication of resistant infections caused by *P. aeruginosa*.

## 1. Introduction

The misuse and abuse of antibiotics are recognized to create selective pressure, resulting in the widespread development of resistant bacterial strains [1, 2]. Antibiotics are also known to kill “good/beneficial” indigenous bacteria, which may have protective role against pathogenic bacteria [3, 4]. Another important point to consider is that antibiotics have been found to be less effective in biofilm-growing bacteria [5].

Facing these limitations of antibiotics, there is an increasing need for the discovery and the development of antimicrobial agents that present novel or unexplored properties to efficiently control and manage bacterial infectious diseases [6]. Inhibition of bacterial virulence and/or biofilm formation by targeting nonmicrobicidal mechanisms are examples of increasingly explored antipathogenic approaches [7–9]. Among opportunistic pathogenic bacteria,* P. aeruginosa*, which produces several virulence factors, is known to be an important human and plant pathogen, responsible for various infections, particularly in immunocompromised persons [10]. Besides this, the remarkable ability of* P. aeruginosa* to form biofilms in many environments renders antibiotic treatments inefficient and therefore promotes chronic infectious diseases [5, 11].

Three global nonmicrobicidal strategies have been proposed to struggle against pathogenic bacteria with biofilm formation ability by (i) avoiding microbial attachment to a surface; (ii) disrupting biofilm development and/or affecting biofilm architecture in order to enhance the penetration of antimicrobials; and (iii) affecting biofilm maturation and/or inducing its dispersion and degradation [8, 12, 13].

The present review covers the scope of natural compounds from both prokaryote and eukaryote organisms that have been identified to disrupt biofilm lifestyle cycle in* P. aeruginosa* without affecting directly bacterial viability. As a prerequisite and for a better understanding of the proposed mechanisms of action of some of the identified compounds, relevant key molecular actors in* P. aeruginosa* biofilm formation and its regulation, such as the chemical signalization machinery involved in bacteria-environment interaction, including quorum sensing (QS) pathways, will be summarized.

## 2. Biofilm Lifestyle Cycle of* P. aeruginosa*

Biofilm formation is an endless cycle, in which organized communities of bacteria are encased in a matrix of extracellular polymeric substances (EPS) that hold microbial cells together to a surface [14, 15]; these are thought to be determinant in 65–80% of all microbial infections [16–18]. In this microscopic world, biofilms are metaphorically called a “city of microbes” [19, 20] with EPS, which represents 85% of total biofilm biomass, as “house of the biofilm cells” [21]. EPS is composed mainly of biomolecules, exopolysaccharides, extracellular DNA (eDNA), and polypeptides that form a highly hydrated polar mixture that contributes to the overall structural scaffold and architecture of the biofilm [22–24].

**Figure 1:**
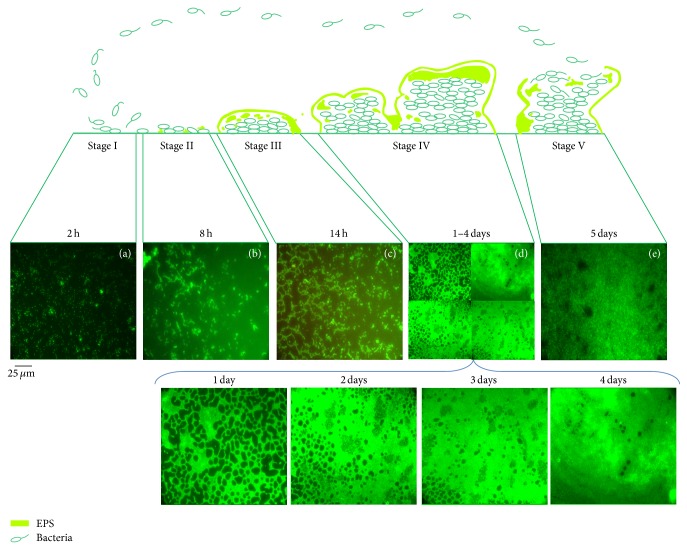
Biofilm lifestyle cycle of* P. aeruginosa* PAO1 grown in glucose minimal media. In stage I, planktonic bacteria initiate attachment to an abiotic surface, which becomes irreversible in stage II. Stage III corresponds to microcolony formation. Stage IV corresponds to biofilm maturation and growth of the three-dimensional community. Dispersion occurs in stage V and planktonic bacteria that are released from the biofilm to colonize other sites. The biofilm formation by* P. aeruginosa* PAO1 was revealed with Syto9 and visualized in Leica DM IRE2 inverted fluorescence microscope with 400x magnification at 2 h (Stage I), 8 h (Stage II), 14 h (Stage III), 1 to 4 days (Stage IV), and 5 days (Stage V). Images represent a 250 × 250-*μ*m field.

Depending on* P. aeruginosa* strains and/or nutritional conditions, different biofilm phenotypes can be developed [25]. For instance, in glucose minimal media, biofilm lifestyle cycle of* P. aeruginosa* PAO1 can be subdivided into five major phenotypic steps (Figure 1). The process begins by the reversible adhesion of planktonic bacteria onto a surface suitable for growth (Figure 1(a), Stage I), followed by irreversible attachment of bacteria, which thereafter form microcolonies in EPS matrix (Figure 1(b), Stage II). Progressively, bacterial microcolonies expand and their confluences lead to a more structured phenotype with noncolonized space (Figure 1(c), Stage III). Then, noncolonized spaces are filled with bacteria, which finally cover the entire surface (Figure 1(d), Stage IV). Meanwhile, the growth of three-dimensional communities is observed (Figure 1, Stages III and IV). Finally, bacteria disperse from the sessile structure and reenter in planktonic state to spread and colonize other surfaces [15, 26] (Figure 1(e), Stage V).

*P. aeruginosa* produces at least three polysaccharides (alginate, Pel, and Psl) that are determinant for the stability of the biofilm structure [27, 28]. Mucoid and nonmucoid* P. aeruginosa* strains differ by the qualitative composition of their polysaccharides in the biofilm matrix, predominantly alginate or Psl/Pel, respectively [29–31]. Alginate, a linear unbranched polymer composed of D-mannuronic acid and L-guluronic acid [32], contributes to the structural stability and protection of biofilms as well as to the retention of water and nutrients [33]. The Pel polysaccharide is mainly a glucose-rich matrix material, with still unclarified composition [34, 35], while Psl comprises a repeating pentasaccharide consisting of D-mannose, L-rhamnose, and D-glucose [36]. Pel and Psl can serve as a primary structure scaffold for biofilm development and are involved at early stages of biofilm formation [30, 37, 38].

eDNA constitutes an important functional component of* P. aeruginosa* biofilm matrix; indeed (i)* P. aeruginosa* biofilm formation is prevented by exposition to DNase I [39]; (ii) biofilms that are deficient in eDNA have been shown to be more sensitive to the detergent sodium dodecyl sulfate [40]; (iii) eDNA facilitates the twitching motility-mediated biofilm expansion by maintaining coherent cell alignments [41]; (iv) eDNA has been proposed to play an important role in the initial and early development of* P. aeruginosa* biofilms as a cell-to-cell interconnecting compound [24, 42, 43]; and (v) finally, eDNA constitutes a nutrient source for bacteria during starvation [44, 45].

Beyond their role in bacterial motilities [46–48],* P. aeruginosa* extracellular appendages flagella, type IV pili and cup fimbriae, are also considered to be matrix components that play adhesive roles in the cell-to-surface interactions (irreversible attachment) as well as in microcolony formation in biofilms. Mutants defective in flagellar-mediated motility and mutants defective in biogenesis of the polar-localized type IV pili do not develop microcolonies compared to the wild type strains [49–51].

## 3. Overview of Global Regulating Systems Involved in* P. aeruginosa* Biofilm Formation

**Figure 2:**
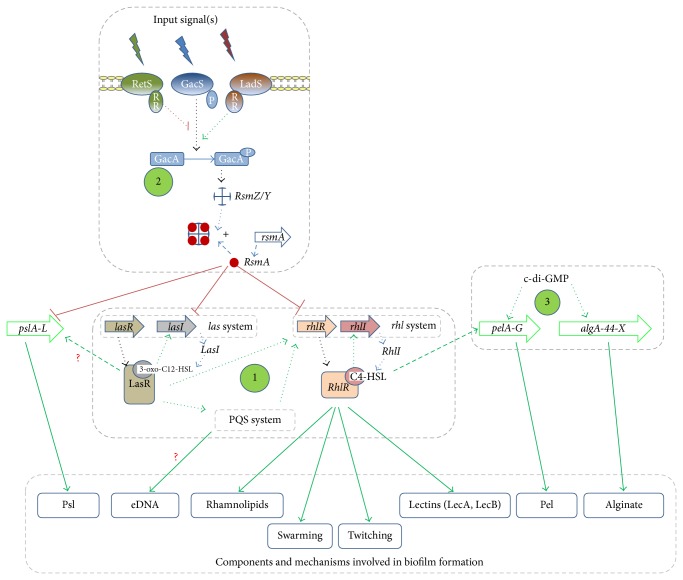
Relevant bacterial systems and factors implicated in the regulation of* P. aeruginosa* biofilm formation. (1) Quorum sensing system; (2) Two-component regulatory system* GacS*/*GacA* and* RetS*/*LadS* (RR: response regulator domain receiver; P: phosphorylation) pathway; (3) Exopolysaccharides production and c-di-GMP pool regulation. See text for explanation.

The complex regulation of biofilm formation involves multiple bacterial machineries, including the QS systems and the two-component regulatory systems that both interact mainly with EPS production [52]. Deficiency in the network regulation required for biofilm matrix formation effectively results in the alteration of the biofilm structure and architecture and, therefore, of its protective role. The main key actors relevant in the regulation of biofilm formation by* P. aeruginosa* are summarized in Figure 2.

### 3.1. QS Mechanisms and Biofilm Formation

**Figure 3:**
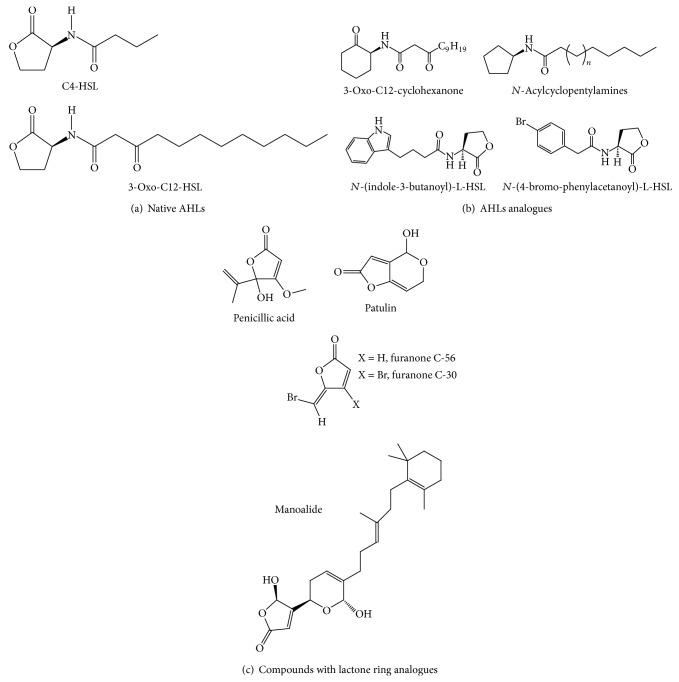
Structure of natural and synthetic AHL-based compounds which inhibit biofilm formation by* P. aeruginosa*. (a) Native* N*-acyl-l-homoserine lactone, signal molecules of* P. aeruginosa* (C4-HSL and 3-oxo-C12-HSL), (b) synthetic analogue of AHLs with side aromatics and synthetic analogues of AHLs with modified lactone rings, and (c) natural (manoalide, penicillic acid, and patulin), and synthetic (furanones) compounds with lactone ring analogues.

QS is a cell-to-cell communication used by many bacteria to detect their population density by producing and perceiving diffusible signal molecules that coordinate virulence factors production, motility, and biofilm formation [53, 54].* P. aeruginosa* possesses two main QS systems (*las* and* rhl*) which drive the production (throughout synthases LasI and RhlI) and the perception (by the transcription factors LasR and RhlR) of the autoinducer signaling molecules* N*-(3-oxododecanoyl)-L-homoserine lactone (3-oxo-C12-HSL) and* N*-butanoyl-L-homoserine lactone (C4-HSL) (Figure 3(a)), respectively [54]. A third QS system, based on quinolone signals (PQS system), interacts with the acyl homoserine lactones (AHLs) systems in an intricate way [54].

Davies et al. [55] have evidenced the role of the* las* system for biofilm formation and maturation; compared to wild type biofilm, the biofilm of* lasI* mutant appears flat, undifferentiated, and quickly dispersed from the surface upon exposure to sodium dodecyl sulfate. The precise implication of* las* system in biofilm formation is not yet clear. However, Gilbert et al. [56] reported that the QS regulator LasR can bind to the promoter region of the* psl* operon, suggesting that QS can regulate* psl* expression. The* rhl* system has been reported to intervene in* P. aeruginosa* biofilm formation [57] by enhancing Pel polysaccharide biosynthesis; transcription of the* pel* operon is actually reduced in* rhlI* mutant. The PQS system, for its part, is linked to eDNA release during biofilm development; biofilm formed by* pqsA* mutant contains less eDNA than biofilm formed by the wild type [40, 42]. All together these data indicate that the three QS systems known in* P. aeruginosa* play roles in biofilm lifestyle cycle.

Importantly, an indirect link between biofilm formation and QS has been reported, through the control of swarming and twitching motilities, as well as rhamnolipids and lectins production. The swarming motility, a form of organized surface translocation, depends on extensive flagellation and cell-to-cell contact [58, 59]; regulated by the* rhl* system [60], swarming motility is implicated in early stages of* P. aeruginosa* biofilm establishment. Strains grown under conditions that promote swarming motility (growth medium with glutamate or succinate as carbon source) form flat and uniform biofilm while strains with limited swarming motility result in biofilm containing nonconfluent cell aggregates [25]. Twitching motility, a flagella-independent form of bacterial translocation, occurs by successive extension and retraction of polar type IV pili [47]. Known to be regulated by the* rhl* system on Fe-limited minimal medium [61], twitching motilities are necessary for the assembly of a monolayer of* P. aeruginosa* cells into microcolonies [49].

Beyond their biosurfactant and virulence factor roles [62], rhamnolipids, whose production is under the* rhl* system control [63], present multiple roles in biofilm formation by* P. aeruginosa*. Indeed, they are believed to be involved in (i) forming microcolonies [64]; (ii) maintaining open channel structures that prevent bacterial colonization by disrupting both cell-to-cell and cell-to-surface interactions [26]; (iii) facilitating three-dimensional mushroom-shaped structures formation in* P. aeruginosa* biofilms [64]; and (iv) facilitating the cell dispersion from the biofilm as* P. aeruginosa* variants which produce more rhamnolipids than wild-type* P. aeruginosa* exhibit hyper-detaching properties [65, 66]. Finally, the cytotoxic virulence factor, galactophilic lectins LecA and LecB, has been proposed to contribute to biofilm development in* P. aeruginosa*, since LecA and LecB mutants form thin biofilms as compared to the wild type bacteria [67, 68]. Both LecA and LecB expressions are regulated by the* rhl* QS system [69].

### 3.2. Biofilm Regulation by* GacS*/*GacA* and* RetS*/*LadS* Two-Component Systems

Among the 60 two-components systems found in the genome of* P. aeruginosa* [70], the* GacS*/*GacA* system acts as a super-regulator of the QS system and is involved in the production of multiple virulence factors as well as in biofilm formation [71]. The Gac system consists of a transmembrane sensor kinase (GacS) that, upon autophosphorylation, transfers a phosphate group to its cognate regulator (GacA) which in turn upregulates the expression of the small regulatory RNAs (*RsmZ* and* RsmY*).* RsmZ* and* RsmY* capture the small RNA-binding regulatory protein RsmA (encoded by* rsmA* gene), a repressor that posttranscriptionally regulates the* psl* locus (*pslA*-*L*) [72–74]. The* GacS*/*GacA* system also has a control on the AHL system as it inactivates free RsmA which negatively controls the synthesis of C4-HSL and 3-oxo-C12-HSL and therefore the extracellular virulence factors controlled by the* las* and* rhl* systems [75–77].

The hybrid sensor histidine kinase RetS is known to repress biofilm formation [78, 79] whereas the histidine kinase LadS antagonizes the effect of RetS [80]. Indeed, Δ*retS* mutant form more structured biofilms as compared to wild type* P. aeruginosa* PAO1 [78]; the PA14 strain (naturally deficient in* ladS* gene) displays attenuated biofilm formation compared to PA14* LadS* ^+^ strain [81]. It is reported that RetS and LadS interact with the* GacS*/*GacA* system by modulating the phosphorylation state of GacS, which consequently inhibits and promotes, respectively, the phosphorylation of GacA [82, 83].

It is interesting to note that* GacS*/*GacA* and* RetS*/*LadS* systems are proposed to be involved in mediating the transition of the* P. aeruginosa* phenotype from an acute to chronic phase infection [78].

### 3.3. C-di-GMP-Dependent Polysaccharides Biosynthesis and Biofilm Formation

Polysaccharides production is dependent on the intracellular pool of bis-(3′-5′)-cyclic dimeric guanosine monophosphate (c-di-GMP) [84, 85], a ubiquitous intracellular second messenger widely distributed in bacteria [86]. In bacterial cells, c-di-GMP is generated from two molecules of guanosine triphosphate by diguanylate cyclases and broken down into 2-GMP by specific phosphodiesterases [86].

High levels of c-di-GMP promote the biosynthesis of polysaccharides (alginate and Pel). Indeed, a binding process of c-di-GMP to PelD and Alg44 proteins is required for Pel and alginate polymer formation, respectively [85, 87]. However, the exact molecular mechanism by which this interaction regulates the polymerization of sugar precursors is not known.

Conversely, low levels of c-di-GMP promote bacterial motilities by enhancing flagellar formation and bacterial dispersion [85].

## 4. Natural and Synthetic Products That Affect* P. aeruginosa* Biofilm Formation

Plants and animals are naturally exposed to bacterial infections and they respond to bacterial components and signal molecules in different manners, including the activation of defense mechanisms and/or the expression of stress management genes [88–93]. Therefore, it is obvious to expect that eukaryotes have developed chemical mechanisms to combat pathogens by killing them or silencing virulence mechanisms such as QS system and/or biofilm formation. Tables 1 and 2 summarize the reported natural and synthetic products that affect* P. aeruginosa* biofilm formation.

### 4.1. Antibiofilm Compounds with Anti-QS Activity

Several classes of molecules have been reported to present both antibiofilm formation and anti-QS properties in* P. aeruginosa* [94–96].

Some AHL analogues (Figure 3(b)) have been shown to exhibit this double inhibitory activity. Geske et al. [97] have reported that synthetic analogues of AHLs with additional aromatic moieties [*N*-(indole-3-butanoyl)-L-HSL and* N*-(4-bromo-phenylacetanoyl)-L-HSL] display inhibitory activity on LasR-based QS system as well as biofilm formation in* P. aeruginosa* PAO1. Synthetic AHLs analogues, where the homoserine lactone ring is replaced by a cyclohexanone ring, downregulate expression of the* LasI* AHL synthase, resulting in a reduced expression of the virulence factors pyocyanin and elastase and in an alteration of biofilm morphology/phenotype [98]. Nonhydrolysable cyclopentyl analogues of AHLs (*N*-acyle cyclopentylamides) inhibit the* lasI* and* rhlA* expression, the production of virulence factors, including elastase, pyocyanin, and rhamnolipids, and the biofilm formation, without affecting bacterial growth [99].

Halogenated furanones (particularly furanones C-30 and C-56), inspired from natural compounds produced by the marine macroalga* Delisea pulchra*, exhibit biofilm reduction and target the* las* and* rhl* systems in* P. aeruginosa* [55, 100, 101]. Besides, in mouse lungs infected with* P. aeruginosa*, they were found to inhibit bacterial colonization to improve the clearance of bacteria from the host and to reduce the tissue damage [102].

Among the macrolide antibiotics, azithromycin, derived from* Saccharopolyspora erythraea*, has been the most investigated anti-QS antibiotic that presents a strong QS and biofilm inhibitory effect in* P. aeruginosa* [103–105]. Indeed, at subinhibitory azithromycin concentration (2 *μ*g/mL),* P. aeruginosa* produces lower AHL signal molecules and virulence factors [106, 107] suggesting that the observed biofilm inhibition is at least partially due to the reduction of both C4-HSL and 3-oxo-C12-HSL production [108]. Interestingly, azithromycin has been reported to diminish the expression of* GacA* but also* RsmA* at translational level [109], to inhibit the synthesis of alginate [103] and to reduce the three types of motility (swimming, swarming, and twitching) [110].

Penicillic acid and patulin, two secondary fungal metabolites from* Penicillium* species, were shown to effect QS-controlled gene expression in* P. aeruginosa*, most likely by affecting the RhlR and LasR regulatory proteins at posttranscriptional level.* In vitro* studies showed that* P. aeruginosa* PAO1 biofilms treated with patulin and tobramycin were considerably more susceptible to the antibiotic as compared to control biofilms exposed to either tobramycin or patulin alone [111]. However, treatment with patulin alone did not affect development of the biofilm and no hypothesis of mechanisms of action was proposed by authors. The genotoxicity of patulin certainly limits its potential usefulness [112].

Manoalide, a sesterterpenoid from the marine organism* Luffariella variabilis*, exhibits antibiofilm and anti-QS activities (*las* system) in* P. aeruginosa* without bactericidal effects [113], although presenting antibiotic activity against gram-positive bacteria [114].

Solenopsin A alkaloid, isolated from the ant* Solenopsis invicta*, inhibits* P. aeruginosa* pyocyanin production, probably through disruption of the* rhl* signaling system and reduces biofilm production in a dose-dependent manner [115].

Mammalian cells release enzymes called paraoxonases 1 (extracted from human and murine sera) that have lactonase activity; degrading* P. aeruginosa* AHLs, they prevent, in an indirect way, QS and biofilm formation [116, 117].

The phenolic compound curcumin, a major constituent of turmeric roots (*Curcuma longa* L.), downregulates virulence factors (pyocyanin, elastase, and protease) in* P. aeruginosa* PAO1 and inhibits adherence of the bacteria to polypropylene surfaces. This was correlated with a decrease in 3-oxo-C12-HSL production [118]. Rosmarinic acid, a natural phenolic compound produced by the root of* Ocimum basilicum* L. upon* P. aeruginosa* infection, prevents biofilm formation but fails to penetrate mature biofilm under* in vivo* and* in vitro* conditions [89]. Structure-based virtual screenings against LasR and RhlR receptor proteins effectively indicate that rosmarinic acid is a potential QS inhibitor [119]. Ellagic acid derivatives, from* Terminalia chebula* Retz., have been shown to downregulate* lasIR* and* rhlIR* genes expression with a concomitant AHLs decrease, resulting in the attenuation of virulence factor production and in an enhanced sensitivity of biofilm towards tobramycin [120]. Girennavar et al. [121] demonstrated that the furocoumarins from grapefruit juice, bergamottin and dihydroxybergamottin, inhibit the activities of the autoinducers AI-1 (*N*-3 hydroxybutanoyl-homoserine lactone) and AI-2 (furanosyl borate diester) in a* V. harveyi* bioassay. Besides, these authors showed that AI-1 and AI-2 inhibit biofilm formation in* E. coli* O157:H7,* Salmonella typhimurium*, and* P. aeruginosa* without affecting bacterial growth. However, the mechanisms of action remain unclear.

Docking screening for QS inhibitors predicted that the flavone baicalein, obtained from the roots of* Scutellaria baicalensis* Georgi, could interact with* A. tumefaciens* QS transcription activator protein TraR. Effectively, at 20 *μ*M, baicalein promotes the proteolysis of the signal receptor TraR protein in* Escherichia coli* biosensor, significantly inhibiting the biofilm formation by* P. aeruginosa* [122]. Similarly, the screening of traditional Chinese medicinal plants identified the anthraquinone emodin, extracted from rhubarb (*Rheum palmatum* L.); emodin actually inhibits the* P. aeruginosa* biofilm formation at 20 *μ*M, increasing the activity of ampicillin [123].

**Figure 4:**
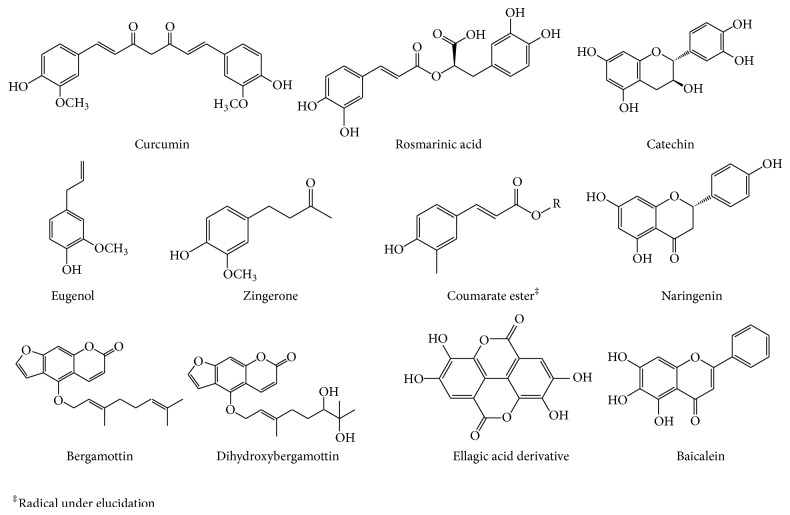
Phenolic compounds and derivatives with antibiofilm and anti-QS proprieties.

**Figure 5:**
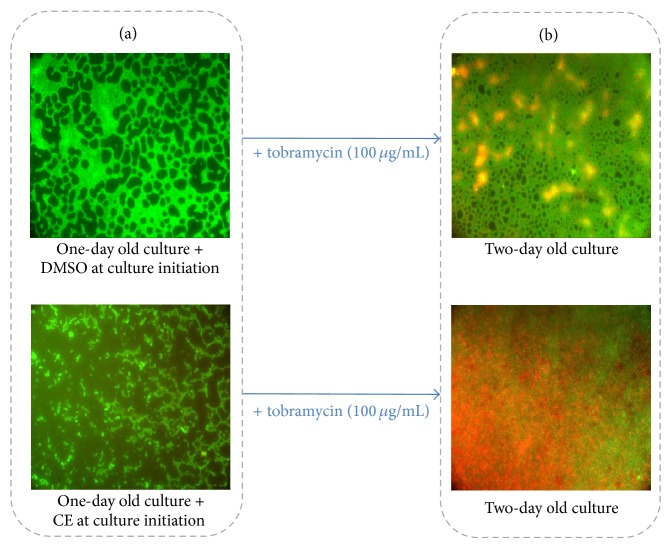
*P. aeruginosa* biofilm phenotypes and effectiveness of tobramycin treatment in presence of DMSO 1% or coumarate ester (CE) at 300 *µ*g/mL. (a) After 1 day of incubation,* P. aeruginosa* fails to form structured confluent aggregate in presence of CE as compared to DMSO treatment. (b) CE considerably increases the susceptibility of* P. aeruginosa* to tobramycin (100 *µ*g/mL), as shown by the increased proportion of dead cells compared with DMSO. The bacterial viability was assessed by staining the cells with SYTO-9 (green areas—live bacteria) and propidium iodide (red areas—dead bacteria) furnished in the LIVE/DEAD* Bac*Light kit. Cells were visualized using a Leica DM IRE2 inverted fluorescence microscope using a 40x objective lens and colored images were assembled using Adobe Photoshop.

The flavan-3-ol catechin, isolated from the bark of* Combretum albiflorum* (Tul.) Jongkind, as well as the flavanone naringenin, both at 4 mM final concentration, do interfere with QS mechanism in* P. aeruginosa* PAO1 by affecting autoinducers perception and biofilm formation [124–126]. A coumarate ester isolated from the bark extract of Malagasy endemic* Dalbergia trichocarpa* Baker interferes with* P. aeruginosa* QS systems (*las* and* rhl*), inhibits the biofilm formation and increases the effectiveness of the antibiotic tobramycin in killing biofilm-encapsulated* P. aeruginosa* [126] (Figures 4 and 5).

Recently,* Meliaceae*,* Melastomataceae*,* Lepidobotryaceae*, and* Sapindaceae*, collected from neotropical rainforests in Costa Rica, presented significant anti-QS activities in a* Chromobacterium violaceum* bioassay and/or inhibition of biofilm formation by* P. aeruginosa* PA14 [127]. Although the exact natures of the active constituents are not yet elucidated, the authors suggest that they could belong to polar polyphenols similar to tannic acid.

A recent screening of various herbal extracts revealed that clove extract (*Syzygium aromaticum* (L.) Merr. Et Perry) inhibits QS-controlled gene expression (*las* and* pqs* systems) in* P. aeruginosa* with eugenol as major active constituent [128]. Eugenol, at subinhibitory concentrations (400 *μ*M) inhibited virulence factors production including elastase, pyocyanin and biofilm formation. In agreement with this finding, subinhibitory concentrations of the clove essential oil significantly reduces* las*- and* rhl*-regulated virulence factors, exopolysaccharide production, and biofilm formation by* P. aeruginosa* PAO1 [129].

Ajoene, an allyl sulfide isolated from garlic (*Allium sativum* L.), has been reported to affect QS-regulated genes in* P. aeruginosa*, including the production of rhamnolipids. Additionally, ajoene synergizes with the antibiotic tobramycin in killing biofilm-encapsulated* P. aeruginosa*, improving the clearance of* P. aeruginosa* from lungs in a mouse model of pulmonary infection [130]. A naturally-inspired organosulfur compound (*S*-phenyl-L-cysteine sulfoxide) and its derivative (diphenyl disulfide) have been reported to significantly reduce the amount of biofilm formation by* P. aeruginosa* [131]. The* S*-phenyl-L-cysteine sulfoxide antagonizes both the* las* and* rhl* QS systems whereas the diphenyl disulfide only interferes with the* las* system.

### 4.2. Antibiofilm Compounds without or with Unspecified Anti-QS Activity

Various organisms, including prokaryotes and eukaryotes (marine organisms, animals, and plants) have been reported to produce secondary metabolites which exert antibiofilm activity. Some of those natural compounds have been used as models to build synthetic antibiofilm compounds against* P. aeruginosa*.

Bromoageliferin, pyrrole-imidazole alkaloids from marine sponges (*Agelas conifer*,* Agelaceae*), has been the scaffolding for the development of two derivatives,* trans*-bromoageliferin analogue 1 (TAGE) and* cis*-bromoageliferin analogue 2 (CAGE). Both synthetic derivatives inhibit biofilm formation and furthermore are able to disperse preexisting* P. aeruginosa* PAO1 biofilms without demonstrating a bactericidal or growth-inhibiting effect [132]. Analogues based upon the oroidin template, parent molecules of bromoageliferin, have been synthesized and screened in* P. aeruginosa* for their antibiofilm ability [133]. The authors found that the most potent analogue turned out to be dihydrosventrin, a variant of the pyrrole-imidazole alkaloids sventrin (from* Agelas sventres*) which exhibits biofilm inhibition and biofilm dispersion for different strains of* P. aeruginosa* without any microbicidal activity.

Alginate lyase, produced by* P. aeruginosa* itself, promotes biofilm dispersion and acts synergically with antibiotics for successful elimination of mucoid strains of* P. aeruginosa* established in the respiratory tracts of cystic fibrosis patients [134]. However, a recent study demonstrated that this effect cannot be attributed to the catalytic activity of the enzyme. Indeed, bovine serum albumin or simple amino acids lead to the same results. The authors postulate that alginate lyase acts simply as a nutrient source, modulating cellular metabolism and thus inducing cellular detachment and enhancing tobramycin efficacy [135].

Bovine pancreatic Dnase I and Dnase-1L2, extracted from human* stratum corneum*, exhibited strong antibiofilm activity in* P. aeruginosa* [136]. Indeed, the degradation of extracellular DNA leads to an altered biofilm that permits increased antibiotics penetration [137].

Extracts of Ginger (*Zingiber officinale* Rosc.), long used by Indians, Asians, and Arabs to treat numerous ailments [137], inhibit* P. aeruginosa* PA14 biofilm formation through the reduction of c-di-GMP production and consequent reduction of total polysaccharides production [138]. The ginger extract revealed no AHL-based QS inhibition in the* Chromobacterium violaceum* CV026 and* Agrobacterium tumefaciens* NT1 reporter biosensor systems. The major component of dry ginger root, zingerone (vanillyl acetone), has been shown to inhibit biofilm formation, to increase the susceptibility of* P. aeruginosa* PAO1 to ciprofloxacin [139] and to inhibit swimming, swarming, and twitching motilities. However, authors did not propose any mechanism of action.

The casbane diterpene, isolated from the ethanolic extract of* Croton nepetaefolius* Baill., a plant native from northeastern Brazil, inhibits biofilm formation in several clinical relevant species, including* P. aeruginosa* (at 250 *μ*g/mL) without affecting the planktonic growth. Authors suggest that this inhibition of biofilm formation may be related to an interaction between casbane diterpene and lipopolysaccharides present on the cell surface, which might affect their adherence properties [140].

Ursolic acid (3*β*-hydroxy-urs-12-en-28-oic acid) from* Diospyros dendo* Welw. is identified to inhibit biofilm formation without interfering with QS systems in* E. coli*,* P. aeruginosa,* and* V. harveyi*; ursolic acid, at 10 *μ*g/mL, has been found to reduce 72% of* E. coli* JM109 biofilm. Transcriptomic analyses led to the conclusion that ursolic acid inhibits biofilm formation by inducing motility [141]. The* 3β* -*O*-*cis*-*p*-coumaroyl-20 *β* -hydroxy-12-ursen-28-oic acid, isolated from the same plant, strongly inhibits biofilm formation by* P. aeruginosa* PAO1 [142]. However, the mechanism of activity was not investigated.

## 5. Concluding Remarks and Perspectives

There is increasing evidence that biofilm-mediated infection facilitates the development of chronic infectious diseases and recurrent infections [143–145]. Relevance in using antibiofilm compounds is based on the restoration of antibiotic effectiveness by facilitating their penetration through compromised biofilm structure. Moreover, a degradation of the biofilm matrix could render infectious bacteria reachable to immune defenses (e.g., polymorphonuclear leukocytes, innate, and specific antibodies) [146, 147]. Thus, antibiofilm compounds could be interesting antibiotic adjuvants to prevent or treat chronic infections. Similarly, relevance in using anti-QS compounds is based on the concomitant drastic reduction of virulence factors expression, which gives the necessary time for immune defense systems to elaborate appropriate responses by the recruitment of immune cells and production of specific antibodies. Unlike antibiofilm compounds, anti-QS compounds are interesting to prevent or jugulate acute infection. However, it should also be noted that (i) anti-QS and antibiofilm compounds may lose their appeal in immune compromised patients who often harbor bacteria that are still alive but present in a disorganized and less virulent stage; (ii) QS systems do not control the totality of virulence factors expression; and (iii) the development of anti-QS bacterial resistance cannot be excluded [148]. These facts partly explain why the discovery of QS modulators has not yet led to major therapeutic breakthroughs. In our opinion, such bioactive compounds will probably not substitute antibiotics but rather optimize the effectiveness of infectious diseases treatment, notably through biofilm disruption and antibiotic dose reduction; their use is also appealing to optimize the use of microbicidal products by reducing biofilm encroachment on biomaterials and medical devices.

In the perspective of therapeutic application, very few studies have been progressed to clinical trial. To the best of our knowledge, garlic is the only extract with anti-QS and antibiofilm to have been tested in a clinical trial with nonsignificant results, contrary to its drastic* in vitro* bioactivity effect [149]. One reason of this fact is that behavior of clinical isolates may be different when grown in laboratory condition and in human body which could lead to unexpected biofilm development. Thus, before progressing in clinical trial of relevant bioactive compounds, effort on the improvement of experimental* in vitro* and* in vivo* conditions should be addressed and clinical trial protocols should be discussed.

Potent antibiofilm agents are considered interesting if they exert a sustainable bioactivity; this can be indicated by an activity that resists accumulating bacterial toxins, enzymes, and metabolites for more than 48 h in culture media. As less than half of bioactive products have been tested up to 48 hours, further investigations are warranted to select those compounds with sustained activities, which would have more chances to be active in clinical conditions. Halogenated furanones have been widely studied for their powerful anti-QS and antibiofilm activities (<10 *μ*M) [100]. However, their toxic and carcinogenic properties relegate them so far to the role of positive QS inhibitory controls in laboratory experiments [150, 151]. In this regard, herbal phenolic compounds and their derivatives, frequent in food components, and more particularly those already present in popular and approved herbal drugs (i.e., rosmarinic acid in* Melissa officinalis* L.), are promising candidates to develop antibiofilm agents; however, structure-activity studies are still required to better assign essential structural features responsible for antibiofilm activity. In the same perspective, searching for compounds active at nanomolar levels should be privileged as these could presumably present lower toxicity risks. The QS system is an obvious target for biofilm-associated infections as QS interacts, directly and/or indirectly, in different steps of biofilm formation. Intriguingly, even if QS inhibition is the most extensively studied approach against* P. aeruginosa*, several anti-QS natural compounds have not been yet investigated for their antibiofilm activity (e.g., human sexual hormones and some antibiotics at subinhibitory concentration, notably ceftazidime and ciprofloxacin) [103, 152]. Attractive therapeutic agents are those which modulate QS system(s) with an extending or particular impact on biofilm lifestyle; they could then be helpful as a preventive or curative approach and at every step of infectious diseases (acute and chronic). However, finding universal antibiofilm compounds represents a challenge as biofilm lifestyle, composition, and phenotype strongly depend on several parameters, such as nutritional conditions. In this regard, we support the hypothesis that compounds which target* GacS*/*GacA* pathway are worthy of interest with respect to the pathway hierarchically upstream position that controls positively both QS system and exopolysaccharides biosynthesis (Psl) (Figure 2). Such compounds could possibly impair almost all the biofilm lifestyle cycle of* P. aeruginosa*, from irreversible attachment to dispersion stages (Table 3) and could be powerful allies for conventional antibiotics in the struggle against bacterial biofilm-mediated infections [8, 12, 95].

## Figures and Tables

**Table 1:** Natural inhibitory compounds for *P. aeruginosa* biofilm formation.

Natural products compounds	Origin	Class	Activities				References
			QS inhibition	Biofilm inhibition^(a)^	Dispersion promotion	Synergy with antibiotic	
Alginate lyase	*P. aeruginosa *	Enzyme	−	+	+	+^(1)^	[134]
Ursolic acid	*Diospyros dendo *Welw.	Triterpenoid	−	+ (24 h) motility	NC	NC	[141]
*p*-Coumaroyl-hydroxy-ursolic acid	*Diospyros dendo *Welw.	Coumarate ester of triterpene	NC	+ (24 h)	NC	NC	[142]
Zingerone	*Zingiber officinale *Rosc.	Phenolic compound	NC	+ (168 h) c-di-GMP	NC	+^(2)^	[138, 139]
Casbane diterpene	*Croton nepetaefolius *Baill.	Diterpenoid	NC	+ (24 h) adherence	NC	NC	[140]
DNase I	Bovine pancreas	Enzyme	NC	+ (18–24 h)	+	+^(3)^	[137]
DNase-1L2	Human *stratum corneum *						[136]
Paraoxonases 1	Human and murine sera	Enzyme (lactonase)	+	+ (24 h)	NC	NC	[116, 117]
Manoalide	*Luffariella variabilis* (Polejaeff, 1884) (marine organism)	Sesterterpenoid	+* (las* system)	+ (24 h)	NC	NC	[113, 114]
Solenopsin A	*Solenopsis invicta* (insect; ant)	Alkaloid	+ (*rhl system) *	+ (24 h)	NC	NC	[115]
Catechin	*Combretum albiflorum* (Tul.) Jongkind	Flavonoid	+ (*las rhl systems) *	+ (24 h)	NC	NC	[124]
Naringenin	Commercial	Flavonoid	+ (*las rhl systems) *	+ (48 h)	NC	NC	[125]
Coumarate ester	*D. trichocarpa *Baker.	Phenolic compound	+ (*las rhl systems) *	+ (48 h)	+	+^(1)^	[126]
Ajoene	*Allium sativum *L.	Organosulfur	+ (*las rhl systems) *	+ (96 h)	NC	+^(1)^	[130]
Ellagic acid derivatives	*Terminalia chebula* Retz.	Phenolic compound	+ (*las rhl systems) *	+ (72 h)	NC	+^(1)^	[120]
Rosmarinic acid	*Ocimum basilicum* L.	Phenolic compound	+ (*las rhl systems) *	+ (18 h)	−	NC	[89, 119]
Eugenol	*Syzygium aromaticum* (L.) Merr. Et Perry	Phenylpropanoid	+* (las pqs* systems)	+ (24 h)	NC	NC	[128]
Curcumin	*Curcuma longa *L.	Phenolic compound	+ (AHLs)	+ (48 h)	NC	NC	[118]
Bergamottin and dihydroxybergamottin	*Citrus paradisi* Macfad. (Rio Red and Marsh White grapefruits)	Furocoumarins	+ (AI-1 and AI-2)	+ (24 h)	NC	NC	[121]
Penicillic acid	*Penicillium* species	Furanone	+ (LasR, RhlR)	NC	NC	NC	[111]
Patulin		Furopyranone	+ (LasR RhlR)	—^‡^	NC	+^(1)^	
Emodin	*Rheum palmatum *L.	Anthraquinone	+ (docking traR)	+ (72 h)	NC	+^(3)^	[123]
Baicalein	*Scutellaria baicalensis *Georgi.	Flavonoid	+ (docking traR)	+ (72 h)	NC	+^(3)^	[122]

+: yes; −: no; NC: not communicated.

^‡^Patulin does not affect the development of biofilm.

^(a)^Experiment duration.

^(1)^Aminoglycosides, ^(2)^ciprofloxacin, ^(3)^ampicillin.

**Table 2:** Synthetic inhibitory compounds in *P. aeruginosa* biofilm formation.

Synthetic compounds (Natural compound origin)	Activities				References
	QS inhibition	Biofilm inhibition^(a)^	Dispersion promotion	Synergistic antibiotic and/or immune defense effect	
TAGE and CAGE (Bromoageliferin)	NC	+ (24 h)	+	NC	[132]
Dihydrosventrin (Sventrin)	NC	+ (24 h)	+	NC	[133]
*N*-(4-bromo-phenylacetanoyl)-l-HSL; *N*-(indole-3-butanoyl)-L-HSL (AHLs)	+ AHLs antagonist (*las* system)	+ (24 h)	NC	NC	[97]
3-oxo-C12-cyclohexanone (AHLs)	+ AHLs antagonist (*las* system)	+ (24 h)	NC	NC	[98]
C10-cyclopentylamide (AHLs)	+ (*lasI* and *rhlA*)	+ (24 h)	NC	NC	[99]
Furanone C-30 and C-56 (Furanone)	+ (*las, rhl* systems)	+ (24 h)			[55, 100, 101]
*S*-phenyl-L-cysteine sulfoxide (Cysteine sulfoxide alliin)	+ (*las, rhl systems) *	+ (24 h)	NC	NC	[148]
Diphenyl disulfide (Disulfide derivatives of the alliinase mediated reactions)	+* las system *	+ (24 h)	NC	NC	
Azythromycin^‡^ (Erythromycin)	+ (*gacA, las and rhl systems*)	+ (72 h)	+	+^(1)^	[103, 108, 109]

+: yes; −: no; NC: not communicated.

^‡^At subinhibitory concentration.

^(a)^Experiment duration.

^(1)^Aminoglycosides.

**Table 3:** Factors affecting biofilm lifestyle regulation.

Biofilm lifestyle cycle	Stage I Reversible attachment	Stage II Irreversible attachment	Stage III Maturation-1 (microcolony development)	Stage IV Maturation-2 (maintenance)	Stage V Dispersion
Implicated factors	c-di-GMP	(i) Flagella and type IV pili (ii) Adhesins (iii) Psl, Pel	(i) Cell-to-cell communication *lasI* (ii) Type IV fimbriae (iii) Matrix components: Psl, Pel, eDNA (iv) Lectin A, B (v) *rhlA *	(i) Cell-to-cell communication (ii) Matrix components: Psl, Pel, eDNA (iii) *rhlA *	Rhamnolipids

General target	(i) Promoting planktonic lifestyle (ii) Blocking switch from planktonic to biofilm lifestyle	Reducing initial adhesion and interaction	Interfering with QS communication	(i) Reactivating metabolic activity for antibiotic efficiency. (ii) Interfering with QS communication	Promoting dispersion and degradation

Examples of extracts or compounds	(i) Ginger extract (ii) Ursolic acid	(i) Casbane diterpene (ii) Coumarate ester	Coumarate ester	(i) Solenopsin A (ii) Naringenin (iii) Furanone C-30 and -56 (iv) Flavan-3-ol Catechin (v) Ajoene (vi) Coumarate ester	DNAse I, 1L2
